# El recuento automatizado de células en líquidos biológicos: una revisión

**DOI:** 10.1515/almed-2020-0087

**Published:** 2021-02-11

**Authors:** María José Alcaide Martín, Laura Altimira Queral, Laura Sahuquillo Frías, Laura Valiña Amado, Anna Merino, Luis García de Guadiana-Romualdo

**Affiliations:** Comisión de Magnitudes Biológicas relacionadas con la Urgencia Médica, Sociedad Española de Medicina de Laboratorio (SEQC-ML), Barcelona, España; Comisión de Biología Hematológica, Sociedad Española de Medicina de Laboratorio (SEQC-ML), Barcelona, España

**Keywords:** líquidos biológicos, recuento celular automatizado, recuento en cámara, microscopía óptica

## Abstract

El recuento de células en líquidos biológicos proporciona una información valiosa para el diagnóstico y tratamiento de diferentes patologías. El recuento en cámara y el estudio de la celularidad mediante microscopía óptica han sido consideradas tradicionalmente como método de referencia. Sin embargo, esta metodología implica un tiempo de respuesta del laboratorio elevado, carece de la reproducibilidad adecuada y requiere de personal experto. El avance tecnológico ha permitido el desarrollo de módulos de análisis específicos para los líquidos biológicos, incorporados en analizadores de hematología y de orinas, que permiten la automatización del recuento celular y han sido rápidamente incorporados a la práctica asistencial de los laboratorios En la actualidad diferentes analizadores están disponibles para ofrecer soluciones de automatización en el recuento de células en líquidos biológicos. Sin embargo, el empleo de dichos analizadores no está exento de limitaciones y su utilización requiere de un profundo conocimiento por los especialistas de la Medicina de Laboratorio. En esta revisión, se describen las principales tecnologías para la automatización del recuento celular en líquidos biológicos, el significado de los parámetros informados por los analizadores, sus principales características analíticas, así como el papel de la microscopía óptica en un contexto de utilización creciente de estas tecnologías.

## Introducción

El estudio de los líquidos biológicos representa una importante actividad asistencial para el laboratorio clínico [1]. Dicho estudio comprende magnitudes bioquímicas, citológicas y microbiológicas, que evaluadas conjuntamente son útiles para el diagnóstico de aquellas patologías que causan su acumulación y/o alteración de sus características.

El método de referencia para el estudio citológico de los líquidos biológicos es el recuento manual por personal cualificado en cámara citométrica y la revisión del diferencial mediante microscopía óptica tras la citocentrifugación y tinción del espécimen [2]. Esta metodología presenta limitaciones: las condiciones para la preparación de las tinciones varían notablemente entre laboratorios, condicionando su calidad, requiere un tiempo prolongado para su realización que aumenta el tiempo de respuesta intra-laboratorio, presenta una alta imprecisión derivada de la variabilidad inter- e intra-observador y requiere de personal altamente cualificado. Además, la citocetrifugación puede afectar a la recuperación de las células, originando su pérdida o cambios morfológicos, que conlleven errores diagnósticos [3]. Por ello, los resultados obtenidos mediante la microscopía tradicional deben ser evaluados cuidadosamente por sus potenciales fuentes de error [4].

Para dar soluciones a algunas de estas limitaciones, la industria del diagnóstico *in vitro* ha adaptado analizadores destinados al recuento celular en otros fluidos como sangre u orina, para el recuento en diferentes líquidos biológicos extravasculares, contribuyendo a simplificar su análisis, mejorar la precisión del recuento y la estandarización del mismo [5].

Pero el empleo de estos analizadores requiere también por parte del especialista de Medicina de Laboratorio de un conocimiento profundo de los mismos, incluyendo sus limitaciones, que en ocasiones obligan a recurrir a la metodología tradicional. Así, un primer paso es el requerimiento de realizar una verificación previa de las especificaciones del analizador para estos especímenes [3], [6].

## Materiales y métodos

### Estrategia de búsqueda y criterios de selección

Se llevó a cabo una búsqueda bibliográfica en la base de datos de PubMed, sin limitación respecto al período de tiempo y sin restricciones de idioma. Para seleccionar solamente estudios en los que el rendimiento del recuento automatizado fuera evaluado frente al recuento en cámara citométrica y diferencial mediante el método de referencia, la microscopía óptica, se utilizó la siguiente estrategia de búsqueda, combinando los términos siguientes: body fluid AND cell count AND (automated OR automatization) AND/OR microscopy. Además, se buscó literatura gris utilizando el motor de búsqueda de Google.

Durante el proceso de selección, dos autores de la revisión evaluaron de forma independiente todos los documentos obtenidos mediante la estrategia de búsqueda. Tras examinar los títulos y resúmenes para eliminar los estudios no relacionados, se recuperó el texto completo de todos los registros restantes. Se resolvieron los desacuerdos mediante la discusión y se consultó a un tercer autor de la revisión cuando fue necesario.

## Resultados

### La automatización del recuento de los líquidos biológicos

Tecnologías ya incorporadas a los analizadores hematimétricos y de orina han demostrado que pueden ser utilizadas para el recuento celular en los líquidos biológicos. Las magnitudes informadas habitualmente por estos analizadores son:

– Recuento total de células nucleadas (TNC-BF), término recomendado por el *Clinical and Laboratory Standards Institute* (CLSI) [7]
– Recuento total de leucocitos (WBC-BF)
– Recuento de hematíes (RBC-BF)
– Recuento de células polimorfonucleares (PMN-BF) y de células mononucleares (MN-BF), expresados en porcentaje y valor absoluto
– Además, algunos analizadores proporcionan magnitudes, sólo con fines de investigación, de otras poblaciones leucocitarias o informan sobre la presencia de otro tipo de células.

### Analizadores hematológicos

#### Analizadores Sysmex (*Roche Diagnostics*)

Diferentes series de los analizadores hematológicos Sysmex, comercializados en España por Roche Diagnostics, disponen de un módulo dedicado (“XN-BF *mode*”), aprobado por la *Food and Drug Administration* (FDA)*,* para el recuento de células en diferentes líquidos biológicos, y que utiliza la citometría de flujo con fluorescencia para el TNC-BF y WBC-BF y la impedancia para el RBC-BF. Desde los años 2011 y 2015, respectivamente, se comercializan las series XN y XN-L, aunque son numerosos los estudios realizados con analizadores de las series XT y XE, disponibles todavía en muchos laboratorios [8], [9], [10]. Aquellos, además del TNC-BF, WBC-BF y RBC-BF, proporcionan el PMN-BF y MN-BF y, como parámetros de investigación, entendidos como aquellos parámetros informados por el analizador pero no validados para su uso clínico, el recuento de neutrófilos (NE-BF), eosinófilos (EO-BF), linfocitos (LY-BF), monocitos (MO-BF) y células de alta fluorescencia o “*HF-cells”* (HF-BF), además de un RBC-BF con mayor sensibilidad analítica. Estos analizadores permiten disminuir el tiempo de respuesta de laboratorio, requieren un bajo volumen de espécimen (88 μL para la serie XN y 70 μL para la serie XN-L), disponen de material de control de calidad para el módulo de líquidos biológicos, no requieren del tratamiento previo de la muestra, excepto en el caso del líquido sinovial [11], disponen de un sistema de alarma para la presencia de células atípicas, útil como criterio para la revisión mediante microscopía óptica, presentan un límite de cuantificación adecuado para el recuento en líquidos de baja celularidad, como el LCR, y proporcionan un recuento diferencial con cuatro poblaciones, cuya transferibilidad ha sido recientemente evaluada [12].

Diversos estudios de evaluación de los analizadores de la serie XN y XN-L han confirmado su utilidad como una alternativa práctica y fiable al recuento manual [12], [13], [14], [15], [16], [17]. Aunque algunos estudios describen una tendencia a la sobreestimación del recuento de algunas poblaciones celulares [13], [15], estas diferencias no son clínicamente significativas, dado el alto grado de acuerdo (95%) del recuento de leucocitos obtenido por ambos métodos para la clasificación de los líquidos [15], siendo el grado de concordancia entre ambos métodos superior en muestras con baja celularidad [8], [15]. Un estudio reciente evaluando la utilidad de estos analizadores para el estudio de la celularidad en líquidos pericárdicos obtenidos en pacientes sometidos a cirugía cardiaca ha confirmado su valor para el recuento de células totales, pero cuestiona su utilización para el recuento diferencial, dada la falta de transferibilidad de los resultados, que los autores atribuyen a la composición peculiar de este líquido, en comparación con otros líquidos serosos, con un elevado porcentaje de células mesoteliales, con tendencia a agregarse; por ello, los autores recomiendan la microscopía óptica como el método más fiable para su estudio [18].

Respecto a la imprecisión, diversos estudios [11], [15], utilizando muestras de LCR, han reportado una imprecisión en equipos de la serie XN inferior al 20%, criterio recomendado para este tipo de ensayo [19], [20] para WBC-BF, TNC-BF, PMN-BF y MN-BF a bajos recuentos (<10 células/μL), e inferior al 10% para recuentos más elevados. Utilizando los materiales de control específicos para el módulo de líquidos biológicos, la imprecisión para ambos niveles es inferior al 10% para el WBC-BF, PMN-BF y MN-BF [13], hallazgo también descrito en analizadores de la serie XN-L [21]. Otras características analíticas de estos analizadores se recogen en la Tabla 1.

**Tabla 1:** Características analíticas de los analizadores hematológicos y Glocyte para el análisis de líquidos biológicos.

Referencia	Líquidos	LB	LD	LC	Arrastre	Linealidad	
		(en células/μL)					
Analizadores Sysmex serie XN							

[8]	LCR, LAs, LPl, LPerit	No evaluado	No evaluado	WBC-BF: 5,0	<0,05%	WBC-BF r^2^=0,99 (se observó un sesgo medio en el rango bajo de recuentos [5–12/μL] de −22,68% con una recuperación media del 81,8%) RBC-BF r^2^=0,99 (las diferencias entre ambos recuentos fueron mayores en el rango 0–200/μL)^a^	
[11]	LSi	TNC-BF: 0,9	TNC-BF: 2,0	TNC-BF: 2,6	0,00%	TNC-BF: r=1,0 (rango: 43–46718/μL)	
		WBC-BF: 0,6	WBC-BF: 1,6	WBC-BF: 2,9		WBC-BF: r=1,0 (rango: 43–46688/μL)	
		PMN-BF: 0,6	PMN-BF: 1,6	PMN-BF: 22,7		PMN-BF: r=1,0 (rango: 41–40216/μL)	
		MN-BF: 0,0	MN-BF: 1,1	MN-BF: 9,1		MN-BF: r=1,0 (rango: 2–6854/μL)	
[15]	LCR	TNC-BF: 0,7	TNC-BF: 1,6	TNC-BF: 3,0	<0,1%	TNC-BF r=1^b^ WBC-BF r=1^b^	
		WBC-BF: 0,3	WBC-BF: 1,2	WBC-BF: 3,0			
		MN-BF: 0,2	MN-BF: 1,6	MN-BF: 6,0			
		PMN-BF: 0,3	PMN-BF: 1,3	PMN-BF: 8,0			

Analizadores Sysmex serie XN-L							

[17]	LCR, LAs, LPl y otros (incluyendo LPerit, LPe y LSi)	RBC-BF: 0 TNC-BF: 0 PMN-BF: 0 MN-BF: 0	LCR:	LCR:	<0,06%	LCR:	R^2^=1
			RBC-BF: 200	RBC-BF: 6100		RBC-BF: 1000–4800	
			TNC-BF: 1,0	TNC-BF: 5,4		TNC-BF: 9–4147	
			PMN-BF: 1,4	PMN-BF: 8,2		PMN-BF: 7–3312	
			MN-BF: 0,9	MN-BF: 3,9		MN-BF: 9–1203	
			LPl:	LPl:		LPl:	R^2^=1
			RBC-BF: 500	RBC-BF: 1900		RBC-BF: 100–7900	
			TNC-BF: 1,8	TNC-BF: 8,9		TNC-BF: 8–4191	
			PMN-BF: 1,0	PMN-BF: 8,0		PMN-BF: 9–3304	
			MN-BF: 1,3	MN-BF: 10,6		MN-BF: 10–2457	
			LAs:	LAs:		LAs:	R^2^=1
			RBC-BF: 400	RBC-BF: 2400		RBC-BF: 2000–8100	
			TNC-BF: 1,0	TNC-BF: 3,5		TNC-BF: 5–5571	
			PMN-BF: 0,8	PMN-BF: 8,7		PMN-BF: 9–3103	
			MN-BF: 1,2	MN-BF: 9,0		MN-BF: 12–1753	

Analizador BC-6800 (Mindray)							

[30]^c^	LAs, LPl, LPerit	WBC-BF: 3,0	WBC-BF: 8,0	WBC-BF: 8,0	<0,05%	No evaluada	
[31]^c^	LCR	TNC-BF: 0,0	TNC-BF: 3,0	TNC-BF: 4,0	<0,3%	TNC-BF: 4–1902/μL; r^2^=1,00 WBC-BF: 4–1902/μL; r^2^=1,00	
		WBC-BF: 0,0	WBC-BF: 3,0	WBC-BF: 6,0			
[32]^c^	LAs, LPl	TNC-BF: 1,0	TNC-BF: 3,0	TNC-BF: 4,0	0,00%	TNC-BF: 8–3965/μL; r^2^=0,99	
		WBC-BF: 1,0	WBC-BF: 3,0	WBC-BF: 3,0		WBC-BF: 8–3936/μL; r^2^=0,99	
				PMN-BF: 22,0		PMN-BF: 24–3063/μL; r^2^=0,99	
				MN-BF: 12,0		MN-BF: 18–2279/μL; r^2^=0,99	
[35]^c^	LSi	TNC-BF: 6,0	TNC-BF: 15,0	TNC-BF: 15,0	<0,3%	TNC-BF: 42–29234/μL; r=0,97	
		WBC-BF: 6,0	WBC-BF: 16,0	WBC-BF: 16,0		WBC-BF: 42–29221/μL, r=0,96	
				PMN-BF: 16,0		PMN-BF: 40–25421/μL; r=0,98	
				MN-BF: 23,0		MN-BF: 2–3975/μL; r=0,92	

Analizador serie Cell-Dyn Sapphire (Abbott Diagnostics)							

[38]	LCR, LAs, LPl, LPerit	WBC-BF: 2,3	No evaluado	WBC-BF: 20	0,16% ^d,e^	WBC-BF: 5–900/μL; r^2^=1	
		RBC-BF: 0		RBC-BF: 3000		RBC-BF: 3000–90000/μL; r^2^=1	

Analizador Unicel DxH 800 (Beckman Coulter)							

[21]	LCR, LPl, LAs, LPerit, LSi, LRA	TNC-BF: 12	TNC-BF: 18	TNC-BF: 37	No evaluado^d^	TNC-BF: 20–89000/μL^e^	
		RBC-BF: <1000	RBC-BF: <1000	RBC-BF: >5000		RBC-BF: 1000–6200000/μL^e^	

Analizador GloCyte (Advanced Instruments Inc.)							

[44]	LCR	TNC-BF: 0,47	TNC-BF: 1,2	TNC-BF: 2,6	No evaluado	No evaluado	
		RBC-BF: 0,73	RBC-BF: 0,8	RBC-BF: 2,0			
[45]		TNC-BF: <1	TNC-BF: 1	TNC-BF: 3		Rango de medida analítica TNC-BF: 3–123/μL RBC-BF: 2–123/μL	
		RBC-BF: <1	RBC-BF: 1	RBC-BF: 2			

LB, límite de blanco; LD, límite de detección; LC, límite de cuantificación (recuento celular más bajo que puede ser informado con un coeficiente de variación ≤20%); LPerit, líquido de diálisis peritoneal; LCR, líquido cefalorraquídeo; LAs, líquido ascítico; LPl, líquido pleural; LSi, líquido sinovial; LRA, Líquido de lavado broncoalveolar; TNC-BF, recuento de células totales; WBC-BF, recuento de leucocitos; PMN-BF, recuento de células polimorfonucleares; MN-BF, recuento de células mononucleares; RBC-BF, recuento total de hematíes. ^a^Sensibilidad analítica (según datos de la casa fabricante), 1000/μL. ^b^Rango de recuentos en el que se evaluó la linealidad (para WBC-BF, 6–785 en el laboratorio 1 y 1–640 en el laboratorio 2, y para TNC-BF, 6–787 en el laboratorio 1 y 1–654 en el laboratorio 2). ^c^Datos no disponibles respecto a las características del recuento de hematíes (RBC-BF). ^d^La casa fabricante recomienda siempre realizar un ciclo de lavado con diluyente previo al procesamiento del espécimen. ^e^Según datos de la casa fabricante.

La experiencia acumulada con estos equipos ha generado un conocimiento amplio sobre posibles interferencias en el recuento celular. Así, la presencia de levaduras puede interferir en los WBC-BF, TNC-BF y HF-BF, generando un patrón característico en el escatergrama (“*blue surfboard pattern”*) [22], de ahí la importancia de una revisión sistemática del mismo. Similarmente, pueden observarse falsos incrementos del WBC-BF en el LCR de pacientes oncológicos tratados con Depocyt, quimioterápico para el tratamiento de la meningitis neoplásica; en este caso, este tipo de espécimen no debe ser analizado con contadores automatizados [23].

#### Analizadores Advia (*Siemens Healthineers*)

Los analizadores Advia 2120/2120i disponen de una metodología multifluido (*Unified Fluids Circuit*) para el estudio del LCR. Esta tecnología permite el recuento de RBC-BF, WBC-BF y de PMN-BF, MN-BF, NEU-BF, LY-BF y MO-BF, expresados en valor absoluto y porcentaje. También disponen de una aplicación específica para líquidos biológicos, aprobada por la FDA, que proporciona los TNC-BF y RBC-BF en líquido pleural, ascítico, de diálisis peritoneal y sinovial, previamente tratado con hialuronidasa [24].

Según los datos de la aprobación por la FDA, el límite de detección de estos analizadores es adecuado para el procesamiento de los líquidos serosos, la imprecisión es inferior al 20%, incluso en rangos bajos de celularidad, el arrastre es inferior al 0,1% y los estudios de linealidad confirman que las desviaciones son inferiores al 10% en los rangos estudiados. Además, se dispone de material de control de calidad específico para líquidos biológicos. Sin embargo, son varias las limitaciones de estos analizadores:

– El análisis del LCR requiere una dilución y pretratamiento previo de la muestra, lo que contribuye a aumentar el tiempo de respuesta de laboratorio.
– Aunque los estudios de transferibilidad han confirmado que este equipo es una alternativa al recuento manual en los líquidos ascítico y pleural [24], [25], [26], esa no ha sido confirmada en el líquido de diálisis peritoneal [27]. Además, la correlación demostrada entre el RBC-BF utilizando este autoanalizador y el obtenido mediante citometría de flujo como técnica de referencia es baja (r=0,545) [28].
– No dispone de un mecanismo de alarma de la presencia de células atípicas.
– El recuento de leucocitos puede ser erróneo en LCR con RBC-BF>1500/μL [28], cifra rebajada por algunos autores hasta 250/μL [29].

#### Analizador BC-6800 BF (*Mindray Medical International*)

**Figura 1:**
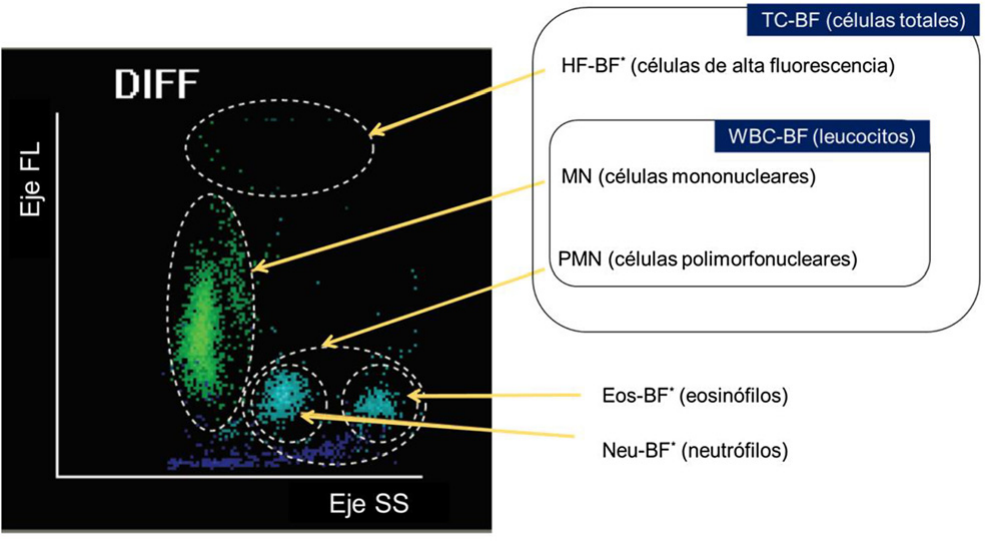
Escatergrama tridimensional característico para la diferenciación (DIFF) de las células nucleadas en el módulo para líquidos biológicos (BF mode) del analizador BC-6800. Las células son agrupadas de acuerdo a su complejidad interna (eje SS), tamaño (eje FS) y contenido en ácidos nucleicos (eje FL). Adaptada de https://www.mindray.com/en/product/BC-6800.html.

El analizador BC-6800 es un analizador hematológico dotado de un módulo específico, no aprobado por la FDA, para el recuento de células en LCR, líquido sinovial y líquidos serosos; recientemente, ha sido también evaluada su utilidad en el líquido de diálisis peritoneal [30]. Dicho analizador proporciona el TNC-BF, WBC-BF, MN-BF y PMN-BF, e informa como parámetros de investigación el recuento de células de alta fluorescencia (HF-BF*), de neutrófilos (Neu-BF*) y eosinófilos (Eos-BF*). El escatergrama que permite evaluar la distribución de la celularidad se muestra en la Figura 1. Este módulo realiza la cuantificación de células nucleadas mediante citometría de flujo con enfoque hidrodinámico (tecnología SF Cube) tras la lisis y tinción fluorescente de las células nucleadas, que son clasificadas en un escatergrama tridimensional de acuerdo con su complejidad interna, tamaño y contenido en ácidos nucleicos. Por otro lado, el analizador dispone de un canal para el RBC-BF en líquidos biológicos mediante impedancia.

Sus principales ventajas son la disponibililidad de un material de control de calidad interno, el corto tiempo para el análisis (<3 minutos), el bajo volumen de muestra requerido (150 μL) y la posibilidad de procesar muestras sin pretratamiento previo.

Estudios recientes han evaluado sus características analíticas (Tabla 1) y la transferibilidad de resultados respecto al método de referencia [25], [30], [31], [32], [33], [34], [35], confirmando su utilidad en LCR [31] y en los líquidos pleural y ascítico [32]. Sin embargo, la evaluación cualitativa sistemática del escartegrama de diferenciación (DIFF) y del HF-BF* es esencial para la generación de algoritmos, como criterios para decidir la necesidad de la revisión microscópica [33]. En este sentido, Buoro et al. [32], [33] recomiendan la revisión en LCR con WBC-BF en el rango entre 4,0 y 7,0/μL, en presencia de un escatergrama DIFF anormal, que puede ser consecuencia de la presencia de microorganismos, con la consecuente repercusión diagnóstica [34], o por incrementos de la diferencia entre el TNC-BF y WBC-BF, que se traduce en el aumento del HF-BF* [31].

En líquido sinovial el recuento celular en el analizador BC-6800 puede reemplazar al análisis mediante microscopía óptica, siendo recomendable el pretratamiento del espécimen con hialuronidasa [35]. Aunque en líquido de diálisis peritoneal, el WBC-BF es transferible al recuento mediante microscopía óptica, la transferibilidad para el recuento diferencial no ha sido evaluada [30].

Hasta nuestro conocimiento, no se han publicado estudios sobre la utilidad de este analizador en el recuento celular en líquido pericárdico.

#### Analizadores de la serie Unicel DxH (Beckman Coulter)

Los analizadores hematológicos Unicel DxH 800/900, capaces de identificar células usando la tecnología *Coulter*, basada en el principio de impedancia, disponen de un módulo específico para el TNC-BF y RBC-BF en líquidos biológicos, utilidad aprobada por la FDA. Además, y como parámetros de investigación, proporcionan un recuento diferencial de las células nucleadas en dos poblaciones, PMN-BF y MN-BF. Similarmente a otros analizadores, también disponen de materiales de control de calidad específicos para este tipo de espécimen.

El estudio reciente de Cho et el. [21] ha confirmado la transferibilidad de resultados del analizador Unicel DxH 800, en comparación con la microscopía óptica, para el TNC-BF y RBC-BF en líquidos serosos y sinovial; sin embargo, en LCR sólo el TNC-BF fue transferible, pero no el RBC-BF, PMN-BF y MN-BF, con desviaciones respecto al método de referencia >20%. Además, en este tipo de líquido, el límite de detección para el TNC-BF fue de 18 células/μL, superior al límite superior de referencia (5 células/μL) recomendado como criterio para el examen microscópico de dicho espécimen [7]. Respecto a la imprecisión, para tres niveles de control, la imprecisión intraserial fue en todos los casos inferior al 10%. Otras características analíticas de este equipo se recogen en la Tabla 1.

#### Analizadores Cell-Dyn (*Abbott Diagnostics*)

Los analizadores de la serie Cell-Dyn, incluyendo CELL-DYN Saphire, son analizadores hematológicos que combina la impedancia y la óptica para el recuento celular, realizándose el recuento diferencial de leucocitos mediante la tecnología de separación por dispersión polarizada multiángulo (*Multi Angle Polarized Scatter Separation*, MAPSS). La limitación principal de este analizador es la no disponibilidad de una aplicación específica para el análisis de líquidos biológicos, además de no disponer de la aprobación por la FDA para este uso. El volumen de muestra requerido es de 120 μL.

A diferencia de otros analizadores hematológicos, la bibliografía publicada respecto a la utilidad de los analizadores Cell-Dyn para el recuento celular de los líquidos biológicos es escasa [25], [36], [37], [38], [39]. En el estudio de De Smet et al. [38] (Tabla 1), evaluando el modelo Sapphire e incluyendo LCR, serosos y de diálisis peritoneal, los autores recomendaron informar sólo WBC-BF y RBC-BF cuando sean superiores a 50/μL y 3000/μL, respectivamente, debido a la elevada imprecisión en recuentos celulares bajos, superior al 80% en recuentos de leucocitos <5/μL, lo que limita su uso en el LCR. Además, este mismo estudio demostró la ausencia de transferibilidad en aquellos líquidos con recuentos por debajo de los límites de cuantificación estimados, así como la incapacidad del analizador para una clasificación diferencial adecuada de los leucocitos en los líquidos serosos, probablemente debida a la clasificación como polimorfonucleares de células mesoteliales y macrófagos. La falta de transferibilidad fue confirmada por Keuren et al. [39].

Este analizador dispone de una alarma para la detección de células con morfología anormal. Un estudio más reciente ha demostrado la capacidad de Cell-Dyn Sapphire para la detección de células tumorales en líquidos serosos, dada la posibilidad de la que dispone este analizador para realizar el inmunofenotipado celular [40].

La línea de analizadores Cell-Dyn va a ser sustituida por el analizador Alinity-hq, con una aplicación específica para líquidos biológicos y cuya utilidad para el recuento de células en sangre ha sido recientemente evaluada [41]. Apenas se dispone de datos sobre su rendimiento en líquidos biológicos; no obstante, evaluaciones internas recientes apuntan a una mejora en la capacidad de detección y en la clasificación de las células, así como a una reducción del volumen requerido [42], [43].

#### Analizador GloCyte (*Advanced Instruments Inc.*)

El analizador Glocyte, recientemente aprobado por la FDA, es un instrumento que proporciona el RBC-BF y TNC-BF en LCR (Tabla 1). A diferencia de los analizadores hematológicos, esta metodología se basa en la detección de la fluorescencia emitida por las células nucleadas, mediante la tinción de los ácidos nucleicos, y los hematíes, mediante el marcaje con anticuerpos marcados con un fluorocromo, utilizando un láser semiconductor y un sistema óptico para capturar imágenes de dichas células. Presenta como ventajas principales el bajo volumen de muestra requerido (30 μL), el corto tiempo de respuesta analítico (5 minutos), la imposibilidad de contaminación por arrastre, debido al empleo de un sistema de cartuchos que impiden el contacto directo del espécimen con el instrumento, la no necesidad de calibración y la disponibilidad de materiales de control de calidad interno. Sus principales limitaciones son que es un analizador diseñado sólo para el procesamiento de LCR, así como la imposibilidad de obtener un recuento diferencial de las células nucleadas, lo que obligaría a recurrir a la metodología tradicional ante recuentos patológicos, la carencia de un sistema de alarma ante la presencia de otros tipos de células, así como el estrecho rango de medida analítica.

Los estudios publicados hasta el momento han demostrado la transferibilidad de resultados para el TNC-BF y el RBC-BF [44], [45]. Respecto a la precisión, este analizador presenta un coeficiente de variación inferior al 20%, para los recuentos de ambos tipos de células; esta precisión se mantuvo por debajo del 20% cuando se analizaron materiales de control y especímenes de pacientes con recuentos próximos a los puntos de corte con relevancia clínica [44].

### Analizadores para urianálisis

#### Analizadores Sysmex

Los analizadores Sysmex (*Sysmex España, SL*) de la serie UF son analizadores de orina basados en el análisis de partículas mediante citometría de flujo con fluorescencia, clasificándose las células en base a tres propiedades: volumen, complejidad interna y contenido en ácidos nucleicos. Las versiones de este analizador (UF-1000i y UF-5000/4000) disponen de un módulo específico para el recuento de células en líquidos biológicos, aunque dicha aplicación no ha sido aprobada por la FDA. Respecto a sus principales características, según datos del proveedor, el límite de detección para ambos analizadores es de 2 leucocitos/μL y 5 hematíes/μL, respectivamente, la muestra no requiere tratamiento previo, el volumen requerido es de 600 μL, una de sus limitaciones más importantes, especialmente en el caso del LCR, y disponen de un sistema de lavado para evitar la contaminación por arrastre. Al igual que para algunos analizadores hematológicos, también se dispone de un material de control de calidad interno. Los estudios de evaluación publicados han demostrado una imprecisión interserial e intraserial para los recuentos de los diferentes tipos de células inferior al 10% y 20%, respectivamente, incluso a bajos recuentos celulares [46], [47]. Las características de estos analizadores descritas en la bibliografía [21], [46], [47], [48] se recogen en la Tabla 2. Es resaltable la variabilidad en los límites de cuantificación obtenidos en los estudios publicados sobre el analizador UF-5000, probablemente debido a diferencias en el método para su estimación.

**Tabla 2:** Características analíticas de los analizadores de orinas para el análisis de líquidos biológicos.

Referencia	Líquidos	LB	LD	LC	Arrastre	Linealidad
		(en células/μL)				
Analizador UF-1000i (Sysmex)						

[46]	LCR	WBC: 0,1	WBC: 0,7	WBC: 2,4	RBC: 0,00%	Comprobada en los intervalos: RBC: 1,9–970/μL (r=1,00) WBC: 0,8–405/μL (r=1,00) Diferencia entre el valor medio y valor esperado ± 10%
		RBC: 1,2	RBC: 5,5	RBC: 18,0	WBC: <0,13%	
[48]	LAs, LPl y LPerit	No evaluado	No evaluado	WBC: 9,2 RBC: 25,0	<0,01%	RBC: r^2^=0,99 WBC: r^2^=1 Intervalos no especificados

Analizador UF-5000 (Sysmex)						

[47]	LCR	TNC: 1	TNC: 1,8	TNC: 1,9	0,00%	Comprobada en los intervalos: RBC: 930–93759/μL (r^2^=0,99) TNC: 3–2957 (r^2^=0,99) WBC: 3–2958/μL (r^2^=1)
		WBC: 1	WBC: 1,8	WBC: 1,9		
		RBC: 2	RBC: 3,5	RBC: 14		
[21]	LCR, LAs, LPl, LPerit, LPe, LSi	TNC: <1	TNC: 2	TNC: 25^a^	No evaluado	No evaluado
		RBC: <1	RBC: <1	RBC: <1^b^		

Analizador Iris iQ2000						

[51]	LCR, LPl, LAs, LPe, líquidos de drenaje	No evaluado	No evaluado	No evaluado	RBC: 0%	RBC: hasta 44000/μL (r^2^>0,9)
					TNC: 0%	TNC: hasta 2600/μL (r^2^>0,9)
[52]	LCR, LAs, LPl, LPerit, LPe	No evaluado	No evaluado	TNC: 35^b^	No evaluado	No evaluado
				RBC: 30^b^		

LB, límite de blanco; LD, límite de detección; LC, límite de cuantificación (recuento celular más bajo que puede ser informado con un coeficiente de variación ≤20%); LPerit, líquido de diálisis peritoneal; LCR, líquido cefalorraquídeo; LAs, líquido ascítico; LPl, líquido pleural; LSi, líquido sinovial; LPe, líquido pericárdico; TNC-BF, recuento de células totales; WBC-BF, recuento de leucocitos; PMN-BF, recuento de células polimorfonucleares; MN-BF, recuento de células mononucleares; RBC-BF, recuento total de hematíes. ^a^LC estimado según el protocolo CLSI EP17-A2. ^b^Denominado LD en el artículo.

La diferencia principal entre ambos analizadores radica en las magnitudes informadas. El analizador UF-1000i proporciona el TNC-BF, WBC-BF y RBC-BF, además de un parámetro de investigación denominado “*Large cells*”, que incluye células mesoteliales, macrófagos y células malignas no hematopoyéticas [46]. Este analizador no proporciona el recuento diferencial de las células nucleadas. El analizador UF-5000 proporciona el TNC-BF, WBC-BF y recuento diferencial, incluyendo células mononucleares (MN-BF) y polimorfonucleares (PMN-BF), expresadas en valor absoluto y porcentual, RBC-BF, “*Epithelial cells*” (EC), magnitud que incluye las células mesoteliales, y bacterias [47], cuya utilidad en la detección e identificación de estos microorganismos como predictor de la positividad del cultivo ha sido recientemente demostrada [49]. Una de las limitaciones principales es la imposibilidad del analizador para informar alarmas que sugieran la necesidad de revisión mediante microscopía óptica.

Diversos estudios han evaluado el grado de concordancia en los recuentos obtenidos con ambos analizadores y el método de referencia en cámara citométrica. En el caso del UF-1000i, Fleming et al. [48] demostraron un buen acuerdo para el RBC-BF, pero solo aceptable para el WBC-BF, de forma similar al demostrado por Buoro et al. [46] en muestras con recuentos <30 células/μL y más recientemente por Maleb et al. [50]. En estos estudios, el analizador sobreestimó el WBC-BF. Aunque el software dispone de una alarma de error para advertir de la presencia de ciertos interferentes, como lípidos, proteínas, restos celulares, bacterias, levaduras y hematíes lisados de forma incompleta, cuya agregación conduce a falsos incrementos del recuento celular, en su estudio Fleming et al. [48] describen incrementos asociados a la presencia de levaduras y bacterias, no advertidos por el equipo, y que se relacionan con la metodología utilizada para el recuento, la citometría de flujo, dado que la agregación de estos microrganismos, que contienen en su estructura pequeñas cantidades de ácidos nucleicos, generarían señales iguales a las de leucocitos y hematíes. Por ello, los autores consideran esencial la revisión sistemática del escatergrama, y en presencia de cualquier alteración detectada, recurrir siempre al análisis microscópico. En el caso del analizador UF-5000, Seghezzi et al. [47] reportaron un buen grado de acuerdo para el RBC-BF; para el TNC-BF y WBC-BF los autores describieron un ligero sesgo positivo (10/μL para WBC-BF y 8,2/μL para TNC-BF), que disminuyó a recuentos <20/μL (1,8/μL para WBC-BF y 2,5/μL para TNC-BF), aunque sin significación clínica. En el reciente estudio de Cho et al. [21] también el acuerdo entre el recuento automatizado y manual de las magnitudes del recuento diferencial de leucocitos (PMN-BF y MN-BF) fue evaluado, observándose para ambos una tendencia a infraestimar dichos recuentos.

#### **Analizador Iris iQ200** (***Iris Diagnostics***)

Los analizadores de la serie IQ 200, comercializados en España por *Beckman Coulter*, disponen de un módulo específico aprobado por la FDA para el TNC-BF y RBC-BF en líquidos biológicos, pero no proporciona el recuento diferencial de células nucleadas. Este analizador usa la tecnología *Digital Flow Morphology*, que permite la cuantificación y caracterización de las células, cuyas imágenes son individualizadas y clasificadas por el software *Auto-Particle Recognition*, permitiendo así la revisión y validación en pantalla y el reconocimiento de células con una morfología anormal. A diferencia de otros analizadores, este equipo requiere de dos diluciones previas del espécimen, siendo el volumen mínimo requerido, en el caso de líquidos de baja celularidad, de 200 μL para líquidos serosos y de 500 μL para LCR, lo cual puede ser una limitación el análisis de este tipo de espécimen [51], [52]. De forma similar a otros equipos, el Iris iQ200 dispone de materiales de control específicos para líquidos biológicos.

Los estudios publicados evaluando la transferibilidad de resultados en comparación con la metodología tradicional han demostrado un buen acuerdo entre ambas metodologías, incluso para TNC-BF<10 μL [51], [52], [53]. Los datos de imprecisión varían en función del estudio y las concentraciones de los especímenes o materiales de control utilizados para su evaluación; así, en el estudio de Butch et al. [53] tanto la imprecisión interserial como intraserial fue ≤10% para el RBC-BF y TNC-BF; sin embargo, en otros estudios [51], [52] los coeficientes de variación fueron superiores al 10%, e incluso más altos que los obtenidos con el recuento manual [51]. Las principales características analíticas del analizador Iris iQ200 se recogen en la Tabla 2.

### Papel del recuento manual de líquidos biológicos en el contexto del recuento automatizado

La introducción en la práctica de los laboratorios clínicos de analizadores para el recuento automatizado de las células en los líquidos biológicos en sustitución del recuento manual es un fenómeno creciente [6].

Sin embargo, la automatización del recuento de células en líquidos biológicos no está exenta de ciertas limitaciones. En primer lugar, no todos los analizadores disponen de la sensibilidad analítica y la precisión adecuada para el recuento de células en fluidos como el LCR [7]. La introducción de nuevas tecnologías probablemente contribuirá a subsanar esta limitación [54]. En segundo lugar, aunque algunos analizadores son ya capaces de informar recuentos diferenciales de 4 poblaciones incluyendo neutrófilos, linfocitos, monocitos y eosinófilos [54], la mayoría de ellos están validados para informar el PMN-BF y MN-BF, lo que contraviene las recomendaciones del *International Council of Standarization in Haematology* (CLSI), que recomienda la inclusión en el informe de todas las células derivadas del sistema hematopoyético y desaconseja el empleo del término “célula mononuclear”, que incluye linfocitos, monocitos, granulocitos inmaduros y blastos [7]. Esta diferenciación cobra gran importancia en situaciones clínicas como el derrame pleural maligno, en el que el cociente neutrófilos/linfocitos se utiliza para establecer el pronóstico del paciente [55]. Por último, los analizadores para el recuento celular automatizado de los líquidos biológicos son incapaces de reconocer, y por tanto de informar, otros tipos de células no hematopoyéticas, grupo en el que se incluyen células de revestimiento de origen diverso, incluyendo las células mesoteliales, blastos, células de linfomas, células derivadas de tumores sólidos y células atípicas; esta celularidad siempre debe ser incluida en el informe de laboratorio, con una descripción morfológica de las mismas

Como se ha descrito anteriormente, algunos analizadores, disponen de un mecanismo de alarma o “*flag*”, denominado “*high-fluorescent cells*” (*HF-cells*). Esta alarma es un indicador de la presencia de células con una elevada relación núcleo-citoplasma y un elevado contenido en ácidos nucleicos y su presencia ha sido propuesta como criterio para la revisión mediante microscopía óptica de la celularidad de la muestra por un experto cualificado, dado que su recuento se ha correlacionado de forma significativa con la presencia de células mesoteliales y/o malignas [33]. Sin embargo, son todavía varias las limitaciones para la implantación de las *HF-cells*, incluyendo la falta de estandarización entre las diferentes metodologías [33] y la falta de consenso respecto a que recuento o porcentaje de las mismas debe ser considerado como criterio para la revisión microscópica manual, probablemente debido a diferencias en los criterios utilizados en los estudios para establecer la positividad de la microscopía óptica y en los objetivos finales de estos estudios, realizados principalmente con analizadores Sysmex [17], [56], [57], [58], [59], [60], [61], [62], [63], [64], [65] (Tabla 3). En el HF-BF, además de células de origen neoplásico, se clasifican otros tipos de células benignas como macrófagos y células mesoteliales, que reducirían la especificidad de esta magnitud para la detección de malignidad [66], con valores que varían entre el 55% y el 87% [61], [62], [63]; la modificación del algoritmo utilizado por el analizador Sysmex-XN 1000 para la detección de *HF-cells* en base a las características de la célula maligna puede ser útil para mejorar la especificidad en la detección de dicho tipo de células [67]. Finalmente, existen factores que condicionan la sensibilidad de esta magnitud [61], [62], [63]; así, el HF-BF varía de forma significativa en función del tipo de tumor, con valores más altos en carcinomas, en comparación con los procesos linfoproliferativos y el mesotelioma, en los que las células malignas no cumplen de forma estricta con los criterios para que la célula sea clasificada como *HF-cell*.

**Tabla 3:** Principales estudios evaluando la utilidad de las *HF-cells*.

Referencia	Líquido	Analizador	AUC ROC	Punto de corte HF-BF^a^	
[17]	LCR, LAs, LPl y otros (LRA, LPerit, LPe, LSi, drenajes, quistes, LAm)	Sysmex XN-550	HF-BF%: 0,79	HF-BF%: 7,9/100 WBCs (S: 64.9%/E: 99.2%)	
			HF-BF#: 0,65	HF-BF#: 46/μL (S: 49.2%/E: 82.0%)	
[56]	LAs	Sysmex XN-1000	HF-BF%: 0,662	HF-BF%: 3,95/100 WBCs (S: 62,1%/E:58,9%)	
			HF-BF#: 0,829	HF-BF#: >17/μL (S: 72,4%/E: 81,9%)	
	LPl		HF-BF%: 0,707	HF-BF%: 4,05/100 WBCs (S:62,5%/E: 71,3%)	
			HF-BF#: 0,730	HF-BF#: >17/μL (S:70,8%/E: 66,2%)	
	LCR		HF-BF%: 0,747	HF-BF%: 0,75/100 WBCs (S:66,7%/E: 79,3%)	
			HF-BF#: 0,717	HF-BF#: >1/μL (S: 33,3%/E: 88,7%)	
[57]	LPl	Sysmex XN-9000	HF-BF%: 0,715	HF-BF%: 5,6/100 WBCs (S: 81,5%/E:52,9%)	
			HF-BF#: 0,663	HF-BF#: 29,5/μL (S: 70,4%/E: 61,8%)	
			HF-BF% + CEA: 0,890	S: 96,3%/E: 73,5%	
			HF-BF# + CEA: 0,860	S: 93,8%/E: 76,5%	
[58]	LPl, LAs	Sysmex XN-9000	HF-BF%: 0,63	No calculado	
			HF-BF#: 0,78	HF-BF#: 68/μL (S: 61%/E: 100%)	
[59]	LCR^b^, LAS, LPl, LPe, LRA, LPerit, LSi	Sysmex XN-9000	HF-BF%: 0,791	HF-BF%: 6,9/100 WBCs (S: 87,2%/E: 60,4%)	
			HF-BF#: No calculado	HF-BF#: No calculado	
[60]	LAs	Sysmex XN-350	TNC-BF#: 0,82	TCN-BF#: >341/μL HF-BF%: No calculado HF-BF#: >28/μL Criterio recomendado para el cribado de carcinomatosis peritoneal: TC-BF# ≥250/μL y HF-BF# ≥17/μL (S: 91%/E: 77%)	
			HF-BF%: 0,55		
			HF-BF#: 0,85		
[61]	LPl, LAs, LCR, LPe	Sysmex XN-1000	HF-BF%: 0,749	HF-BF%: 5,3/100 WBCs^c^ (S: 75%/E: 63%) HF-BF#: 67/μL^c^ (S: 73%/E: 87%)	
			HF-BF#: 0,835	HF-BF%: 2,7/100 WBCs^d^ (S: 92%/E: 40%) HF-BF#: 16/μL^d^ (S: 92%/E: 46%)	
[62]	LAs, LPe, LPerit, LPl	Sysmex XN-2000	HF-BF%: 0,69	HF-BF%: 2,1/100 WBCs (S: 86%/E: 46%) HF-BF#: 17/μL (S: 88%/E: 61%)	
			HF-BF#: 0,77		
[63]	LPl, LAs	Sysmex XN-1000	HF-BF%: 0,707	HF-BF%: 4,4/100 WBCs (S: 79,2/E: 55,8%) HF-BF#: 24,5/μL (S: 75,3%/55,0%)	
			HF-BF#: 0,708		
[64]	LPl	Sysmex XN-350	HF-BF# ≥17/μL (S: 94%/E: 50%) → AUC ROC: 0,718 HF-BF# ≥10/μL (S: 98%/E: 42%) → AUC ROC: 0,70 HF-BF# ≥17/μL (excluyendo pacientes con ICC e infección; S: 91%/E: 79%)→ AUC ROC: 0,849 HF-BF# ≥10/μL (excluyendo pacientes con ICC e infección; S: 94%/E. 74%) → AUC ROC: 0,842 Criterio recomendado para el cribado de derrame pleural maligno: HF-BF# ≥17/μL		
[65]	LAs, LPl	Sysmex XN-1000	Centro 1: HF-BF# ≥108/μL → S: 66,7%/E: 93,6% HF-BF# ≥108/μL + información clínica → S: 100%/E: 68,9%		Centro 2 HF-BF# ≥45/μL → S: 86,8%/E: 66,6% HF-BF# ≥45/μL + información clínica → S: 100%/E: No calculado

LCR, líquido cefalorraquídeo; LAs, líquido ascítico; LPe, líquido pericárdico; LPl, líquido pleural; LPerit, Líquido de diálisis peritoneal; LSi, líquido sinovial; LAm, líquido amniótico; LRA, líquido de lavado broncoalveolar; ICC, insuficiencia cardiaca congestiva; S, sensibilidad; E, especificidad; TNC-BF#, recuento total de células nucleadas, expresado en valor absoluto; HF-BF%, recuento total de *HF-cells*, expresado en porcentaje; HF-BF#, recuento total de *HF-cells*, expresado en valor absoluto. ^a^Punto de corte recomendado como criterio para revisión de la celularidad mediante microscopía óptica o análisis digital de imágenes. ^b^En el análisis por separado de especímenes de líquidos cefalorraquídeos malignos, el HF-BF de *HF-cells* no mostró una correlación significativa con el porcentaje de células malignas mediante microscopía óptica. ^c^Puntos de corte seleccionados para maximizar sensibilidad y especificidad (índice de Youden). ^d^Puntos de corte seleccionado en base a la sensibilidad y valor predictivo negativo, como criterio de exclusión.

No se dispone de datos sobre el rendimiento del HF-BF* en el analizador BC-6800 (Mindray) para la detección de células malignas o como criterio para la revisión mediante microscopía óptica; sólo en el estudio de evaluación de este equipo para el recuento de células en líquidos serosos por Buoro et al. [32] se confirmó la presencia de un recuento superior a 50 *HF-cells*/μL en todas las muestras con un escatergrama anormal, por lo que los autores proponen esta condición como criterio de revisión mediante microscopía óptica.

Algunos de estos estudios proponen algoritmos de trabajo con diferentes criterios para la revisión mediante el examen microscópico, incorporando parámetros de investigación, como las *HF-cells* y el recuento de eosinófilos, o parámetros relacionados con la morfología de los linfocitos [17], [21], [58], [63], [64]. Un estudio reciente concluye que la combinación de la información clínica y el HF-BF mejora de forma significativa la sensibilidad del dato aislado de dichas células [64]

Respecto a los analizadores de orina, en el UF-5000 Sysmex la diferencia entre los recuentos de células nucleadas totales (TNC) y leucocitos (WBC), así como el recuento de “*Epithelial cells*” (EC) puede servir como orientación para la observación mediante microscopía óptica. Sin embargo, en la bibliografía consultada no se han encontrado estudios similares a los realizados en los analizadores hematológicos Sysmex. Un estudio preliminar propone el recuento porcentual de células mononucleares como criterio en la detección de malignidad [68].

### Identificación de las células en líquidos biológicos mediante el análisis digital de imágenes

La utilización de sistemas automatizados de morfología digital facilita y agiliza el análisis de muestras de sangre. Estos sistemas utilizan microscopía motorizada, procesamiento digital de imagen y reconocimiento de patrones para identificar y preclasificar los diferentes tipos de células sanguíneas normales [69]. Uno de los sistemas con mayor implantación en los laboratorios es CellaVision, que en sus versiones DM 96, DM9600, DM1200 y DI-60 dispone de una aplicación para líquidos biológicos que ofrece una preclasificación de las células nucleadas en las siguientes categorías: neutrófilos, eosinófilos, linfocitos, macrófagos (incluyendo monocitos), otras células (basófilos, células de linfoma, blastos y células tumorales) y células no identificadas. Este sistema permite disponer de un sistema experto para la evaluación morfológica de las células en los líquidos biológicos, facilita el reconocimiento de células patológicas y permite reevaluar de forma consensuada con otros expertos dicha morfología.

Diversos estudios han evaluado la utilidad del sistema CellaVision para el estudio de la morfología en los líquidos biológicos [70], [71]. Utilizando la versión DM96, Riedl et al. [70] compararon los resultados en diferentes tipos de líquidos biológicos obtenidos en la post-clasificación de los diferentes tipos de células mediante este sistema con la microscopía óptica, demostrando la transferibilidad de resultados en los líquidos incluidos en el estudio; además la imprecisión para la preclasificación de las células fue inferior al 6% para todos los tipos celulares y el porcentaje de células correctamente clasificadas por el sistema en la preclasificación fue del 90% y 83% en LCR y otros líquidos, respectivamente. Más recientemente, Takemura et al. [71] han evaluado el módulo para líquidos biológicos en el analizador digital de imágenes DI-60 en LCR y líquidos serosos; los resultados de la clasificación de las células mediante este equipo mostraron una buena correlación con el examen microscópico para todas las poblaciones excepto los monocitos, debido a la complejidad morfológica de este tipo de célula.

## Conclusiones

La incorporación a la práctica asistencial de los laboratorios de analizadores capaces de realizar de forma automatizada el recuento celular en líquidos biológicos es una realidad; son varias las ventajas derivadas de su empleo: en la mayoría de los casos no requieren de la preparación previa de la muestra, reducen el tiempo de respuesta del laboratorio y la exposición a riesgos biológicos, suelen requerir un volumen bajo de espécimen y presentan unas características adecuadas para su uso previsto, con bajos límites de detección y cuantificación [19].

Sin embargo, dichos analizadores son una herramienta complementaria, y no alternativa, a la metodología tradicional, basada en la microscopía óptica, y en un futuro, a los sistemas de clasificación de células basados en la imagen digital [20]. La implantación de estos analizadores requiere:

1. Un conocimiento profundo de sus características analíticas, debiendo garantizar una sensibilidad analítica e imprecisión adecuadas para el recuento en líquidos con baja celularidad, especialmente en los recuentos correspondientes a puntos de corte de decisión clínica utilizados para la clasificación del líquido; algunos autores [20] afirman que se ha alcanzado el “techo de cristal” en base a los resultados obtenidos con algunos analizadores [54].
2. Una verificación previa de sus características, de acuerdo al protocolo establecido por la ICSH [6].
3. El uso de equipos que dispongan de un módulo específico para el procesamiento de los líquidos biológicos [4], con un sistema de lavado que evite el fenómeno de arrastre y contaminación por otros especímenes y sin necesidad de tratamiento previo, excepto el líquido sinovial por sus características físicas [11].
4. La evaluación de la imprecisión del recuento celular en líquidos biológicos requiere de materiales de control de calidad específicos para este tipo de espécimen [6], [19].
5. Deben diseñarse algoritmos para el análisis de líquidos biológicos que incorporen criterios para la toma de decisiones respecto a la revisión por personal cualificado [2], [17], [58], [65].

Son varios los retos aún por resolver para el empleo de este tipo de analizadores:

1. Se requieren más estudios para evaluar la utilidad de alarmas como los *HF-cells*, especialmente para el cribado de células malignas. Actualmente, la microscopía óptica es el método de elección para el recuento celular en pacientes oncológicos o sospecha de derrame maligno [56], [59], [61], [62], [63].
2. La industria del diagnóstico *in vitro* debe realizar esfuerzos encaminados al desarrollo de tecnología capaz de cumplir las recomendaciones del CLSI para la evaluación de la morfología celular en los líquidos biológicos [2].
3. Se requieren especificaciones de calidad específicas para valorar el rendimiento de los analizadores para el recuento celular en líquidos biológicos [6].
4. Los laboratorios deben participar en programas externos de control de calidad diseñados para estas magnitudes [6].
5. No se dispone de estudios que hayan evaluado la utilidad del recuento celular automatizado en líquido amniótico.
